# Usage of documented pre-hospital observations in secondary care: a questionnaire study and retrospective comparison of records

**DOI:** 10.1186/1757-7241-21-13

**Published:** 2013-03-01

**Authors:** Geir O Knutsen, Knut Fredriksen

**Affiliations:** 1 Anaesthesia and Critical Care Research Group, Department of Clinical Medicine, Faculty of Health Sciences, University of Tromsø, N-9037, Tromsø, Norway; 2 Division of Emergency Medical Services, University Hospital of North Norway, N-9038, Tromsø, Norway

## Abstract

**Background:**

The patient handover is important for the safe transition from the pre-hospital setting to secondary care. The loss of critical information about the pre-hospital phase may impact upon the clinical course of the patient.

**Methods:**

University Hospital Emergency Care registrars answered a questionnaire about how they perceive clinical documentation from the ambulance services. We also reviewed patient records retrospectively, to investigate to what extent eight selected parameters were transferred correctly to hospital records by clinicians. Only parameters outside the normal range were selected.

**Results:**

The registrars preferred a verbal handover with hand-written pre-hospital reports as the combined source of clinical information. Scanned report forms were infrequently used. Information from other doctors was perceived as more important than the information from ambulance crews. Less than half of the selected parameters in pre-hospital notes were transferred to hospital records, even for parameters regarded as important by the registrars. Abnormal vital signs were not transferred as often as mechanism of injury, medication administered and immobilisation of trauma patients.

**Conclusions:**

Data on pre-hospital abnormal vital signs are frequently not transferred to the hospital admission notes. This information loss may lead to suboptimal care.

## Background

In the recent literature, several authors have addressed the challenges of a safe transfer of responsibility for patients from one team to another [1-9]. For patients seen in emergency medicine, the handover in the Emergency Department (ED) is challenging for several reasons. This multi-professional environment, with different expectations, agendas and communication traditions among the professions [7,10] account for some of the problems. Furthermore, the high workload and continual flow of information is, at times, beyond the limits for human information retention [10,11]. The potential for loss of information continuity is always present and therefore the ED handover is critical for patient safety.

Written clinical documentation is an important supplement to verbal communication. However, hospital records may not necessarily contain clinically relevant information documented by the ambulance staff and handed over by them in the ED [9,12]. The ambulance service patient report form (PRF) contains important documentation of the early phases of care, and because of the dynamic nature of trauma and acute illness this clinical information may be important for the interpretation of clinical findings and treatment strategies after admission.

Occasionally via routine auditing of medical notes, we have observed that critical clinical observations, clearly documented in the PRF, are not transferred to the in-hospital patient record. Sometimes PRF information has been altered when transferred to hospital’s electronic patient record (EPR). For this reason, we decided to investigate the magnitude of missing critical information in patient’s hospital records with respect to what was contained in their PRF. In addition, the Norwegian hospital setting we are working in is different from many other countries, as the ED is staffed with registrars in internal medicine, general surgery and other medical specialities, in contrast to countries where emergency medicine is a separate medical speciality. Our hypotheses were that the hospital clinicians did not regard the PRF as a source of information of importance for hospital treatment, that this was more predominant if the patient was handed over by an ambulance crew and that a significant amount of information about abnormal pre-hospital findings was not transferred to the hospital records.

ED doctors were asked about their perception of the verbal handover and written documentation from both the ground ambulance and physician-manned air-ambulance services. We also studied to what extent selected clinical parameters were correctly transferred from the pre-hospital PRF to the hospital’s EPR.

## Methods

### Study design

The study consists of a cross-sectional questionnaire and retrospective patient record study.

### Setting

The University Hospital of North Norway (UNN) is the regional hospital serving the 478.000 inhabitants of the sparsely populated region of North Norway and the Norwegian Arctic archipelago. In addition, UNN locally serves 120.000 of these inhabitants. Emergency medicine is not an individual medical speciality in Norway. Physicians from the various medical disciplines attend patients admitted to the ED department. The majority of the emergencies are admitted by the ground ambulance service, staffed by personnel with technician or paramedic training. In addition, patients are admitted by a physician-staffed helicopter emergency medical service (HEMS).

Before arrival in hospital, the pre-hospital service will give a brief pre-arrival notification, including vital signs and medications given to the Emergency Medical Communication Centre (EMCC) at the hospital. There are no written guidelines or protocol for the handover from the pre-hospital to the ED personnel. After a verbal handover from the pre-hospital team, the written PRF which documents medical history, vital signs observations, and all treatments administered is given to the ED staff. If a general practitioner (GP) has admitted the patient, the pre-hospital documentation also includes a written note from the GP. When a patient has been admitted by a HEMS physician, the air-ambulance PRF may be supplemented by a more detailed pre-hospital note in the EPR, resembling the traditional EPR notes made by hospital doctors. After admission, an ED nurse will make an admission note in the EPR. Finally, all hand written pre-hospital documents will be scanned, and included in the EPR as picture files. After the handover and subsequent examination of the admitted patient, the attending hospital physician will make a comprehensive admission note in the EPR, concluding with an initial clinical plan for the patient. The verbal handover from pre-hospital personnel is not documented other places than in the admission notes of the receiving physician and ED nurse.

### Questionnaire

We invited all registrars in the departments of internal medicine, surgery and paediatrics at the UNN Tromsø to answer a web-based [13] questionnaire [See Additional file 1]. The questionnaire probed areas such as the respondents’ opinion of the written pre-hospital documentation and verbal handover, as well as their perception of the quality and importance of the pre-hospital information. The answers were given as a score on a scale from 0 to 6. All participants received reminders approximately two weeks apart in order to improve the response rate.

### Inclusion of patients and selection of clinical data

We retrospectively examined all consecutive calls in the electronic records (AMIS) to the UNN Tromsø EMCC classified as code red (or “immediate”) according to the Norwegian Index for Medical Emergencies [14] between 1 June 2009, and 1 February 2010 by which point we had included approximately 500 patients. In order to limit the cases to emergencies, we included only patients with more than one abnormal parameter (see below). Furthermore, we excluded patients not admitted, all patients that were dead on arrival, Code red calls that had been degraded to a lower acuity code, women in labour, and all of the HEMS missions that was given a National Advisory Committee on Aeronautics (NACA) [15] score below 4.

Eight parameters that we considered important for management of the patient were collected from the pre-hospital documentation and compared with corresponding documentation found in the early intra-hospital records. The parameters and normal values were: Respiratory rate (10-20/min), haemoglobin oxygen saturation (≥95%), Glasgow coma score (15), mechanism of injury, oxygen therapy given, fluid therapy given, medications provided and immobilization. Only abnormal values documented or treatment given was recorded for comparison with the intra-hospital records, as these were assumed to be most relevant and important for the intra-hospital course of the patient.

### Data analysis

The data were recorded in a Microsoft Excel® spreadsheet, and simple descriptive statistics and 95% confidence intervals were calculated.

### Approval

The Hospital Data Protection Officer approved the study.

## Results

### Doctor perception of pre-hospital information

64 registrars attending emergency admissions at the UNN Tromsø received the questionnaire and 29 (45.2%) responded. 35% of the respondents worked in the departments of internal medicine, 41% were surgical registrars and 24% were paediatric registrars.

**Figure 1:**
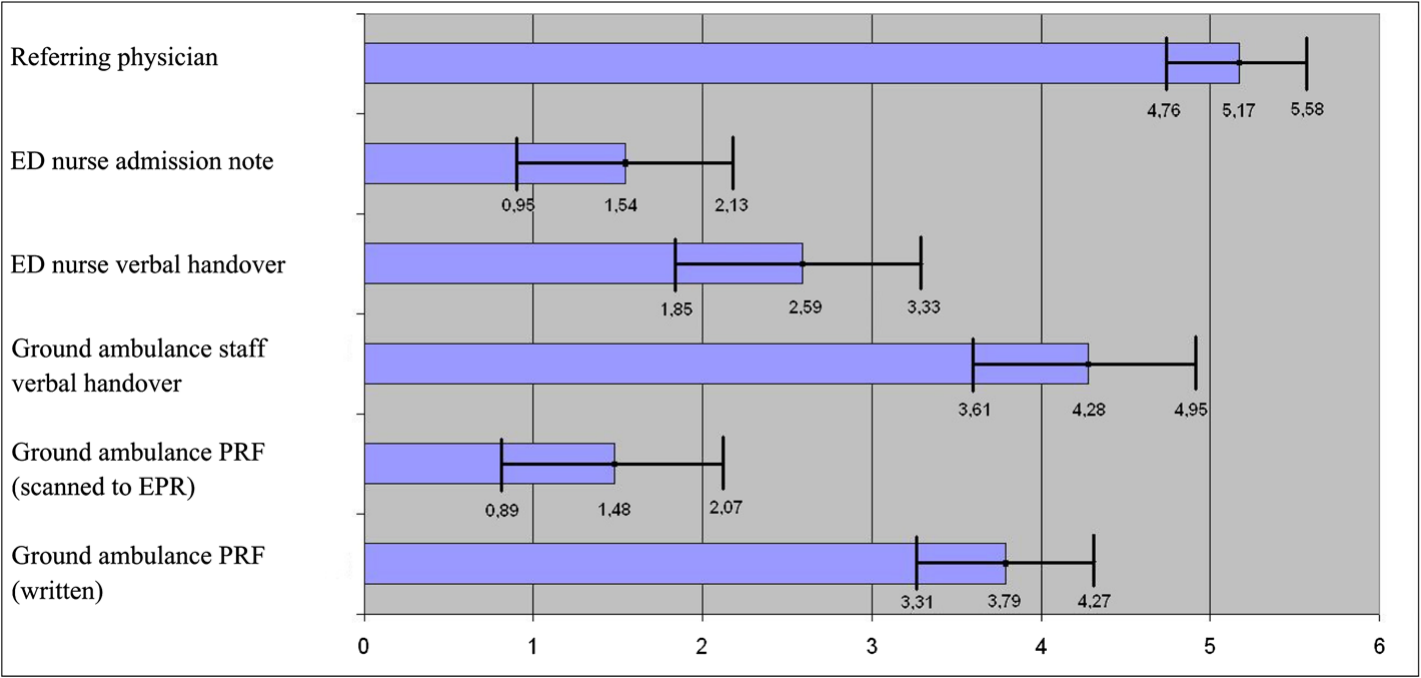
**Preference for patient information – ground ambulance.** The ED physicians’ opinion of pre-hospital information about patients admitted to the ED by ground ambulance. The perceived usefulness was scored on a scale from 0 (not preferred) to 6 (highly preferred).

If the patient arrived by ground ambulance (Figure 1), the two sources for information considered most important was the verbal handover from the ambulance service and, if applicable, the referring GP’s admission note. The only written documentation that received a moderately high score (3.79, on a scale from 0 to 6) was the hand-written PRF from the ambulance staff. However, the scanned PRF was valued significantly lower as a source for pre-hospital information and the ED nurse’s documentation was hardly used at all.

**Figure 2:**
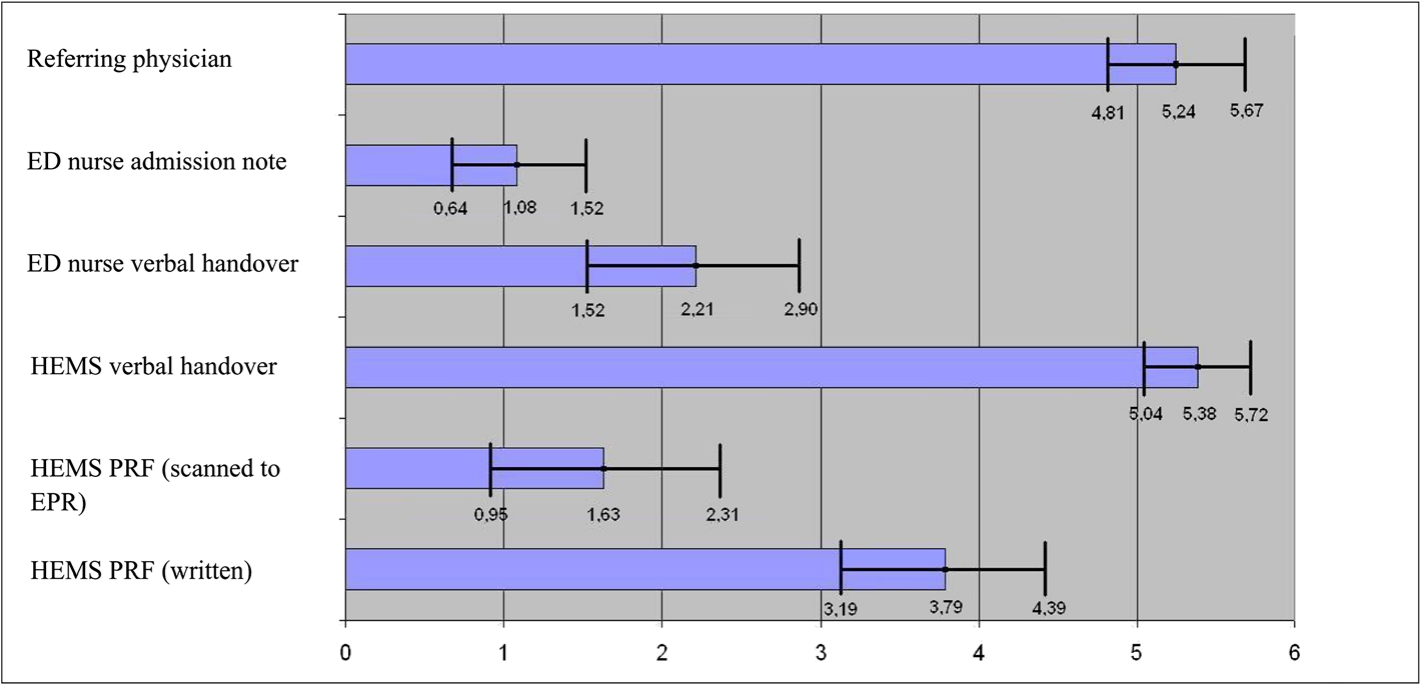
**Preference for patient information – HEMS.** The ED physicians’ opinion of pre-hospital information about patients admitted to the ED by the HEMS. The perceived usefulness was scored on a scale from 0 (not preferred) to 6 (highly preferred).

If the patient had been brought to the ED with the physician-manned HEMS instead of ground ambulance (Figure 2), the verbal handover from the HEMS doctor received the highest score (5.38) of all information sources (both ambulance and HEMS) and the written note from the GP (in these cases) was once again given a high score (5.24). The hand-written HEMS PRF was preferred to the scanned HEMS PRF or the ED nurse’s documentation, suggesting that the attending physicians clearly preferred information from other doctors. Furthermore the verbal handover and the hand-written documents were preferred above scanned files in the EPR.

**Figure 3:**
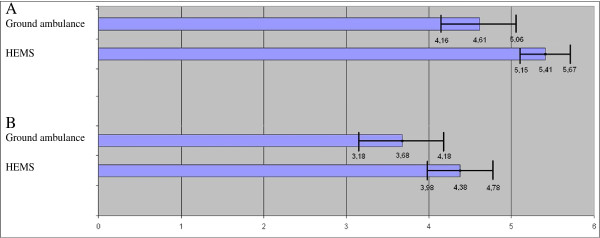
**Perceived importance and quality of pre-hospital data.** (**A**) The extent to which the ED physicians emphasise the pre-hospital observations documented in the pre-hospital records of the ground ambulance and HEMS services when writing the admission note in the hospital patient record, rated from 0 (not preferred) to 6 (highly preferred). (**B**). Perceived quality of pre-hospital information from ground ambulance and HEMS, according to the ED physicians. Rated from 0 (not preferred) to 6 (highly preferred).

Consistent with these findings, the registrars rated air-ambulance observations higher than the observations of the ground ambulance staff and the HEMS observations were emphasised significantly more when writing the EPR admission note (Figure 3).

**Figure 4:**
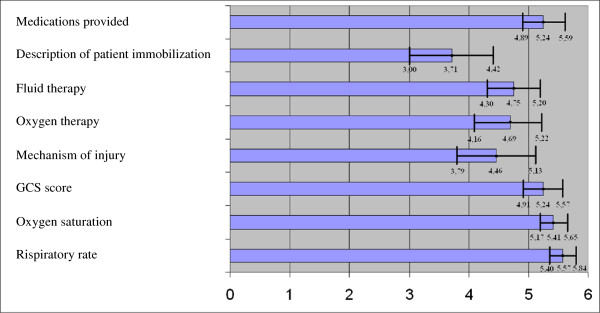
**Perceived importance of different types of pre-hospital data.** ED physicians’ perceived opinion about how important different types of prehospital information documented in the pre-hospital patient records are. Rated from 0 (not important) to 6 (highly important).

As we suspected that the ambulance service staff and hospital doctors might emphasise different variables and observations differently, we questioned the registrars about their opinion of the importance of eight selected variables. Interestingly, an abnormal respiratory rate was the parameter rated highest, with haemoglobin oxygen saturation below 95%, Glasgow coma score (GCS) below 15 and medications administered by the pre-hospital services, rated as the other most important observations in the PRF (Figure 4).

The pre-hospital staff’s evaluation of mechanism of injury for trauma patients, oxygen therapy given, administration of intravenous fluids or immobilisation of trauma patients, seemed less interesting to the hospital doctor. However, the wide confidence intervals for the latter four variables suggested that the doctors may have differed significantly in their opinion, and further analysis did show that surgeons and paediatricians judged mechanism of injury and patient immobilisation data as more important than internists did (data not shown).

### Transfer of pre-hospital information to the patient records

A total of 1750 code red responses initiated by the EMCC in the study period were assessed and 526 patients were eligible for inclusion according to the defined criteria. 25 of the eligible cases were excluded because the PRF was missing in the EPR, leaving 501 cases (379 admitted by ground ambulance, and 122 by HEMS). The pre-hospital records, EMCC records and early intra-hospital EPR notes of the included patients were scanned for the presence of 8 selected parameters. This was done in order to measure the potential loss of clinical information between the ambulance services and the hospital team. 1611 abnormal PRF parameters were recorded.

The first step in the communication between the pre-hospital services and the hospital is the verbal report transmitted by radio or telephone to the EMCC. This information should contain a complete report of vital signs, but also the treatment given and other information important to the receiving hospital. We found that 53% of the selected parameters in the PRF were documented in the AMIS report and the transfer of the individual parameters is shown in Table 1. The EMCC documented mechanism of injury, administered medications and oxygen therapy in the majority of the cases. However, patient immobilisation measures and administration of intravenous fluids were transferred in less than one third of cases. The reason for this may be that the verbal transmission to the EMCC is brief and some of the parameters may be omitted on purpose (i.e. fluid therapy and immobilisation of the trauma patient).

**Table 1:** Transfer of parameters to AMIS

	**PRF**	**AMIS**	
Respiratory rate	226	125	(55.3)
Oxygen saturation	179	84	(46.9)
Mechanisms of injury	47	37	(78.7)
GCS score	149	59	(39.6)
Oxygen therapy	464	255	(55.0)
Fluid therapy	195	54	(27.7)
Medications provided	307	225	(73.3)
Immobilization	44	6	(13.6)

Non-normal parameters recorded in the pre-hospital patient report form (PRF) and transfer to the Acute Medical Information System (AMIS) in the emergency medical communication centre (EMCC) (percent transfer in parentheses). N = 501.

We expected that more data from the written pre-hospital documentation would be transferred into the ED clinicians’ admission note in the EPR. We compared both the ground ambulance PRFs and the HEMS PRF with the EPR.

Rather surprisingly, no more than 30% (317/1058) of the selected parameters recorded in the written ground ambulance PRF were included in the admission note, even though this ambulance document was available to the doctor after the verbal handover and easily accessible as a scanned form in the electronic record later. The transfer of the individual parameters is described in Table 2. Pre-hospital information about the suspected mechanism of injury and medications given was transferred in more than half of the cases, but abnormal vital signs (respiratory rate, haemoglobin oxygen saturation and GCS) were documented in only 12–22% of the cases. Information about immobilisation of trauma victims was more common than vital signs (43% of cases).

**Table 2:** Transfer of parameters to the hospital records: ground ambulance

	**PRF**	**EPR**	
Respiratory rate	184	22	(11.9)
Oxygen saturation	152	20	(13.2)
Mechanisms of injury	21	24	(87.5)
GCS score	104	23	(22.1)
Oxygen therapy	347	74	(21.3)
Fluid therapy	113	13	(11.5)
Medications provided	216	132	(61.1)
Immobilization	21	9	(42.9)

Parameters recorded in the pre-hospital report form (PRF) and in the ED clinician’s admission note in the electronic patient record (EPR). Patients admitted with ground ambulance crew. N = 379.

This trend was also evident for the 122 patients admitted by the HEMS, but data from this physician-manned service were transferred in 44.2% (200/453) of the parameters, compared to only 30% in the ground ambulance patients. This was paralleled by higher figures for the individual parameters (Table 3), and information about mechanism of injury was reproduced in all of the hospital EPR notes. Information about drugs administered and immobilisation was transferred in the majority of the cases, but abnormal vital signs were found less commonly. The only exception was abnormal GCS values recorded by the HEMS doctor, which was transferred to the EPR in 53% of the cases. This finding was clearly higher than for the ground ambulance GCS data (22%).

**Table 3:** Transfer of parameters to the hospital records: HEMS

	**PRF**	**EPR**	
Respiratory rate	42	8	(19.0)
Oxygen saturation	27	7	(25.9)
Mechanisms of injury	26	26	(100.0)
GCS score	104	23	(53.3)
Oxygen therapy	347	74	(21.3)
Fluid therapy	113	13	(11.5)
Medications provided	216	132	(61.1)
Immobilization	21	9	(42.9)

Parameters recorded in the pre-hospital report form (PRF) and in the ED clinician’s admission note in the electronic patient record (EPR). Patients admitted with helicopter emergency medical service (HEMS). N = 122.

## Discussion

We assessed hospital doctors’ perception of the ambulance services’ written records, and the actual transfer of data to the hospital EPR. We selected only parameters outside normal ranges and medical treatment actually provided by the pre-hospital team in order to focus on data with potential importance for the clinical course. We found that less than half of the information was transferred to the attending doctor’s admission note, which is the primary compilation of information about the present medical history and clinical findings in the EPR. This finding is disturbing if it reflects a discontinuity in care of the emergency patient [3,5,6].

Other authors have discussed challenges to good communication between ambulance staff and the ED department clinicians [2]. According to Yong et al. [4] only half of the clinicians referred to the ambulance records, even though they perceived handover information as useful, consistent with the our findings. Thakore and Morrison [10] reported that the ED medical staff found handover quality variable and lacking structure, whereas ambulance crews felt that medical staff did not pay attention to their handovers. Interdisciplinary training in handover skills, development of standardised formats, active listening skills and even electronic transfer of pre-hospital data have been suggested to improve accuracy and completeness in ED handovers [2,4,7,16-19]. Even though standardised communication guidelines to increase patient safety have been recommended by some authors, they are in limited use and positive effects have still not been documented [2]. The usefulness of electronic pre-hospital records has been limited, mostly because they have been difficult to integrate with intra-hospital EPRs [18]. In our hospital, no standardised handover protocol is in use, and the pre-hospital records are not part of the EPR.

Only half of the positive findings documented in the PRF were actually reported in the EMCC records. Still, the fact that information is included in the PRF does not imply that it was transmitted during the brief radio or telephone report before admission. It is probably acceptable that the ambulance crew reduces the amount of information to an appropriate minimum and understandable if EMCC staff omits parts of the transmitted data in their report.

Hospital clinicians preferred the written note from the admitting GP. It is tempting to believe that the clinicians found the structure or format of this note familiar, and resembling the admission note they write in the EPR when compared with the PRF, which is focused on vital signs reporting. It is evident from our results that the doctors emphasise the observations made by the physician manned HEMS significantly more than observations from the technician or paramedic manned ground ambulance service. The notes from the ED nurses was least emphasised of all, probably because they contain only second hand information already conveyed to the clinician by the pre-hospital personnel.

It is interesting to note that the attending doctors prefer the verbal handovers as the main source for information, and not the written or scanned documents. The scanned files are included chronologically with the other notes in the EPR, but the legibility of the picture-file may not be as good as the original document. Thus, the scanned documents may primarily serve as permanent storage of the documentation and to a lesser extent, as a practical source of pre-hospital information. The response rate to the questionnaire was less than 50%, which is disappointing but not an uncommon observation. Web based questionnaires are commonly used in our hospital, and low response rates are often seen, probably because they are considered time consuming. We believe that a selection bias of importance for our study was unlikely if lack of time to participate was the main reason for not answering the survey. However, the possibility of bias cannot be ruled out completely.

The HEMS physicians may chose to write a pre-hospital note in the EPR to document critically important pre-hospital information, advanced treatment, and other information that is considered as important to the hospital staff. This kind of documentation is confined to a limited number of the HEMS patients. Only 8 out of 122 patients in our study had a HEMS note in the EPR. However, we observed that transfer of pre-hospital data to the EPR admission note was more complete in these cases (data not shown). It may theoretically reflect more severe disease or trauma in these 8 patients and the finding may not be explained by the format of the record alone. We still believe that pre-hospital notes written directly into the EPR could represent a format that would be easier to access and preferred as source of information by hospital clinicians.

When analysing the transfer to EPR of the individual parameters we found that information about mechanism of injury and administered drugs were transferred more often than vital signs. It could be that the pre-hospital vital signs observations were found to be unchanged in the ED and thus were considered unnecessary. Nevertheless, the dynamic nature of the pathophysiology of the emergency patient should advocate better documentation of the time course of vital signs.

The clinicians perceived the pre-hospital respiratory rate as the most important parameter, yet only transferred this information in less than 20% of the cases. Respiratory rate carries more prognostic information for trauma patients than any other single parameter [20]. Still it is often not documented in patient records, even for severely traumatised patients [21]. We have focused on better documentation of respiratory rate in our hospital for years, and believe that changes in respiratory rate is important for interpretation of the dynamic changes in the patient’s physiologic state.

Another important observation in our study is that the level of consciousness (GCS) was transferred clearly more often from patients admitted by the HEMS than by ground ambulance. It may be that the patient groups differ with regard to severity and frequency of trauma, but to some extent it may also reflect that a physician-scored GCS was perceived as more significant than GCS scored by ambulance staff. The ED clinicians did not regard information about mechanism of injury as very important in the questionnaire, but these data were almost always transferred to the EPR. This may be explained by the fact that also doctors that not see trauma patients participated in the questionnaire. When we analysed for differences between physicians, paediatricians and surgeons, we found that surgeons emphasised mechanism of injury far more than average.

It has been described earlier that pre-hospital data can be changed during transfer, so that the hospital records contain incorrect information [19]. We have occasionally noted this in our ED department, but this has not been investigated by the present study.

The data that were not transferred from the PRF may represent information with no impact on clinical decisions, and thus our findings may have more medico-legal importance than clinical consequences. Our study design does not allow us to detail this question and this possibility will have to be addressed in future studies. We have raised several issues that should be investigated, for instance the clinical consequences of omitting information, and the flow of verbal information and its use in clinical decisions. However, the findings suggest that there is room for improvement in the handover of emergency patients in our setting.

## Conclusions

We have found that the registrars in our ED prefer the verbal handover and hand-written pre-hospital reports as source of clinical information, and hardly use scanned report forms at all. They also perceive clinical information from other doctors as more important than information from ambulance crews. Less than half of potentially important information documented in available pre-hospital notes is transferred to the hospital records, and we believe this represents a risk for discontinuity in care.

## Abbreviations

AMIS: Acute Medical Information System; ED: Emergency department; EMCC: Emergency Medical Communication Centre; EPR: (hospital) Electronic patient record; GCS: Glasgow coma score; GP: General practitioner; HEMS: Helicopter emergency medical service; NACA: National advisory committee for aeronautics; PRF: (pre-hospital) Patient report form; UNN: University Hospital of North Norway.

## Competing interests

The authors declare that they have no competing interests.

## Authors’ contributions

GOK participated in the design of the study, collected and analysed the data, and wrote the initial draft. KF conceived the study, participated in design and coordination, and helped to draft the manuscript and revised it. Both authors have approved the final manuscript.

## Supplementary Material

Additional file 1 Questionnaire. Click here for file
