# Correction: usage of documented pre-hospital observations in secondary care: a questionnaire study and retrospective comparison of records

**DOI:** 10.1186/1757-7241-22-3

**Published:** 2014-01-22

**Authors:** Geir O Knutsen, Knut Fredriksen

**Affiliations:** 1 Anaesthesia and Critical Care Research Group, Department of Clinical Medicine, Faculty of Health Sciences, University of Tromsø, Tromsø N-9037, Norway; 2 Division of Emergency Medical Services, University Hospital of North Norway, Tromsø N-9038, Norway

## Correction

After the publication of our research article [1], we noticed that incorrect numbers were reported for the five lower parameters in the published Table three (Table 1 here). The published numbers derive from Table two in the article and were mistakenly copied into Table three (Table 1 here). This correction contains the corrected numbers for Table three (Table 1 here).

**Table 1:** Transfer of parameters to the hospital records: HEMS

	**PRF**	**EPR**	
Respiratory rate	42	8	(19.0)
Oxygen saturation	27	7	(25.9)
Mechanisms of injury	26	26	(100.00)
GCS score	45	24	(53.3)
Oxygen therapy	117	36	(30.8)
Fluid therapy	82	18	(22.0)
Medications provided	91	66	(72.5)
Immobillization	23	15	(65.2)

Parameters recorded in the pre-hospital report form (PRF) and in the ED clinician’s admission note in the electronic patient record (EPR).

Patients admitted with helicopter emergency medical service (HEMS). N = 122.
