# Supplementary material for: Usage of documented pre-hospital observations in secondary care: a questionnaire study and retrospective comparison of records
Source: Scand J Trauma Resusc Emerg Med. 2013 Mar 1;21:13. doi: 10.1186/1757-7241-21-13 (PMC3606240; doi:10.1186/1757-7241-21-13)
Supplement: Additional file 1 — Questionnaire. [file 1757-7241-21-13-S1.pdf]

dditional files

uestionnaire

Rate the following seven questions from 0 = not at all, to 6 = to a great extent.

Availability

1.1

If you receive / write admission records in the acute hospitalized patient arriving by ground ambulance, how do you obtain the pre-hospital information if you believe it is important?

Circle the one best suited

Not important	□						
Ground ambulance PRF (written)	0	1	2	3	4	5	6
Ground ambulance PRF (scanned to EMR)	0	1	2	3	4	5	6
Ground ambulance staff verbal handover	0	1	2	3	4	5	6
ED Nurse verbal handover	0	1	2	3	4	5	6
ED Nurse admission note	0	1	2	3	4	5	6
Referring physician	0	1	2	3	4	5	6
Other - please specify:							

1.2

If you receive / write admission records in the acute hospitalized patient arriving from HEMS, how do you obtain the pre-hospital information if you believe it is important?

Circle the one best suited.

Not important	□						
HEMS PRF (written)	0	1	2	3	4	5	6
HEMS PRF (scanned to EMR)		0	1	2	3	4	5
6							
HEMS verbal handover	0	1	2	3	4	5	6
ED Nurse verbal handover	0	1	2	3	4	5	6
ED Nurse admission note	0	1	2	3	4	5	6
Referring physician	0	1	2	3	4	5	6
Other - please specify:							

Quality

2.1

To what extent do you think ground ambulance PRFs contains information of good quality?

Circle the one best suited.

0 1 2 3 4 5 6

2.2

To what extent do you think HEMS PRFs contains information of good quality?

Circle the one best suited.

0 1 2 3 4 5 6

2.3
To what degree do you emphasize the measurements and observations made by the ground ambulance personnel, when you receive the acutely hospitalized patient from ground ambulance?
Circle the one best suited.
0 1 2 3 4 5 6

2.4
To what degree do you emphasize the measurements and observations made by HEMS physician, when you receive the acute hospitalized patient from HEMS?
Circle the one best suited.
0 1 2 3 4 5 6

2.5
Enter importance of the following pre-hospital observations and measures.
Circle the one best suited.

Respiratory rate	0	1	2	3	4	5	6
Oxygen saturation	0	1	2	3	4	5	6
GCS score	0	1	2	3	4	5	6
Mechanism of injury	0	1	2	3	4	5	6
Oxygen therapy	0	1	2	3	4	5	6
Fluid therapy	0	1	2	3	4	5	6
Description of patient immobilization	0	1	2	3	4	5	6
Medications provided	0	1	2	3	4	5	6
